# A Classic Presentation of Acne Keloidalis Nuchae in a Black Male

**DOI:** 10.7759/cureus.85919

**Published:** 2025-06-13

**Authors:** Ami Patel, Micah Pippin, Diahann Marshall

**Affiliations:** 1 Family Medicine, Louisiana State University Health Sciences Center, Alexandria, USA; 2 Family Medicine, Rapides Regional Medical Center, Alexandria, USA

**Keywords:** acne keloidalis nuchae, clinical dermatology, dark skin, family medicine residency, people of color

## Abstract

Acne keloidalis nuchae (AKN), also known as folliculitis keloidalis nuchae and nuchal keloid acne, is a chronic inflammatory skin disorder of the hair follicle. Predominantly occurring in the nuchal and occipital scalp, it can lead to keloid-like plaques and cicatricial alopecia. Primarily affecting people of color, more specifically African American individuals, it is associated with haircuts, friction, or trauma among a variety of other less common causes. The diagnosis is clinical with physical exam findings showing firm, dome-shaped papules or pustules, hypertrophic scars, inflammation, and alopecia. Although challenging as the disease progresses, management includes topical and systemic antimicrobials, corticosteroids, cryotherapy, laser therapy, and surgical excision. Early intervention is vital as it helps minimize the risk of permanent scarring and psychosocial impact. The following case report is about a young, African American male who presented to the outpatient clinic with findings concerning for AKN.

## Introduction

Dermatologic conditions in people of color can present differently, and their prompt identification and management can impact lives drastically. This population, unfortunately, has been underrepresented in dermatology textbooks, research, and clinical trials, leading to inadequate training and education, which in turn leads to underdiagnosed and undertreated skin conditions in people of color. Diagnosing and treating these skin conditions appropriately is vital, as it can improve quality of life and mental health [1]. This case report focuses on one such skin condition that can easily be missed if not identified promptly and correctly. More commonly found in people with Fitzpatrick skin phototypes V and VI, acne keloidalis nuchae (AKN) is defined as persistent folliculitis forming keloid-like scars and cicatricial alopecia usually found on the nuchal and occipital scalp [1-3].

## Case presentation

A 28-year-old African American male presented to an outpatient clinic with complaints of enlarging lesions on the nape of his neck that had been present since his early 20s. The patient reported occasional pruritus; however, denied pain, discharge, drainage, or systemic symptoms, including fever. The patient reported having close-shave haircuts in the past, after which the lesions would appear or worsen. He reported no history of keloids from prior injury, trauma, or scars. Additional medical history included keratoconus of his right eye, for which he had been following up with ophthalmology. He took brimonidine tartrate 0.2% solution ophthalmic drops thrice daily and used fluticasone propionate 50 mcg/actuation intranasally for allergic rhinitis. He did not report any other personal medical history; his only reported surgery was for his eye condition. His family history was non-contributory as there was no reported history associated with AKN, and he was not taking any other prescription or over-the-counter medications. He denied tobacco, alcohol, or illicit drug use and reported no known drug allergies.

**Figure 1:**
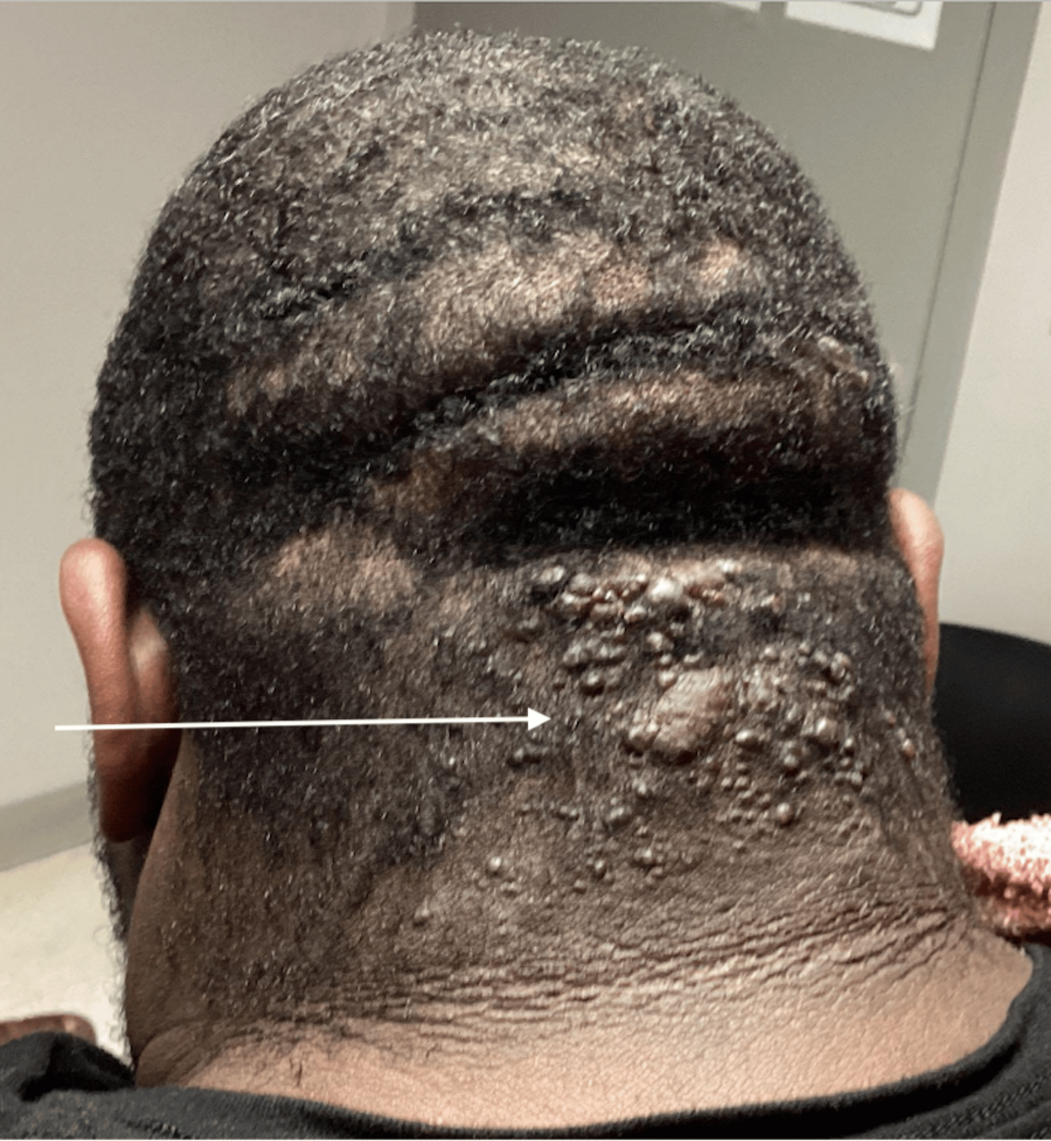
Acne keloidalis nuchae on initial presentation.

During his initial consultation, vital signs were documented as a blood pressure of 141/89 mmHg, a heart rate of 89 beats per minute, a respiratory rate of 16 breaths per minute, and a temperature of 98.5° Fahrenheit (36.9° Celsius). The patient weighed 343 pounds (155.58 kilograms) and had a body mass index of 52.15 kilograms per square meter, making him clinically obese. His Fitzpatrick skin classification was type 6. The physical examination demonstrated well-circumscribed, dome-shaped papules and plaques along the occipital and nuchal scalp, measuring 11 centimeters from the lower edge of the eruption to the highest point of the rash (Figure 1).

**Figure 2:**
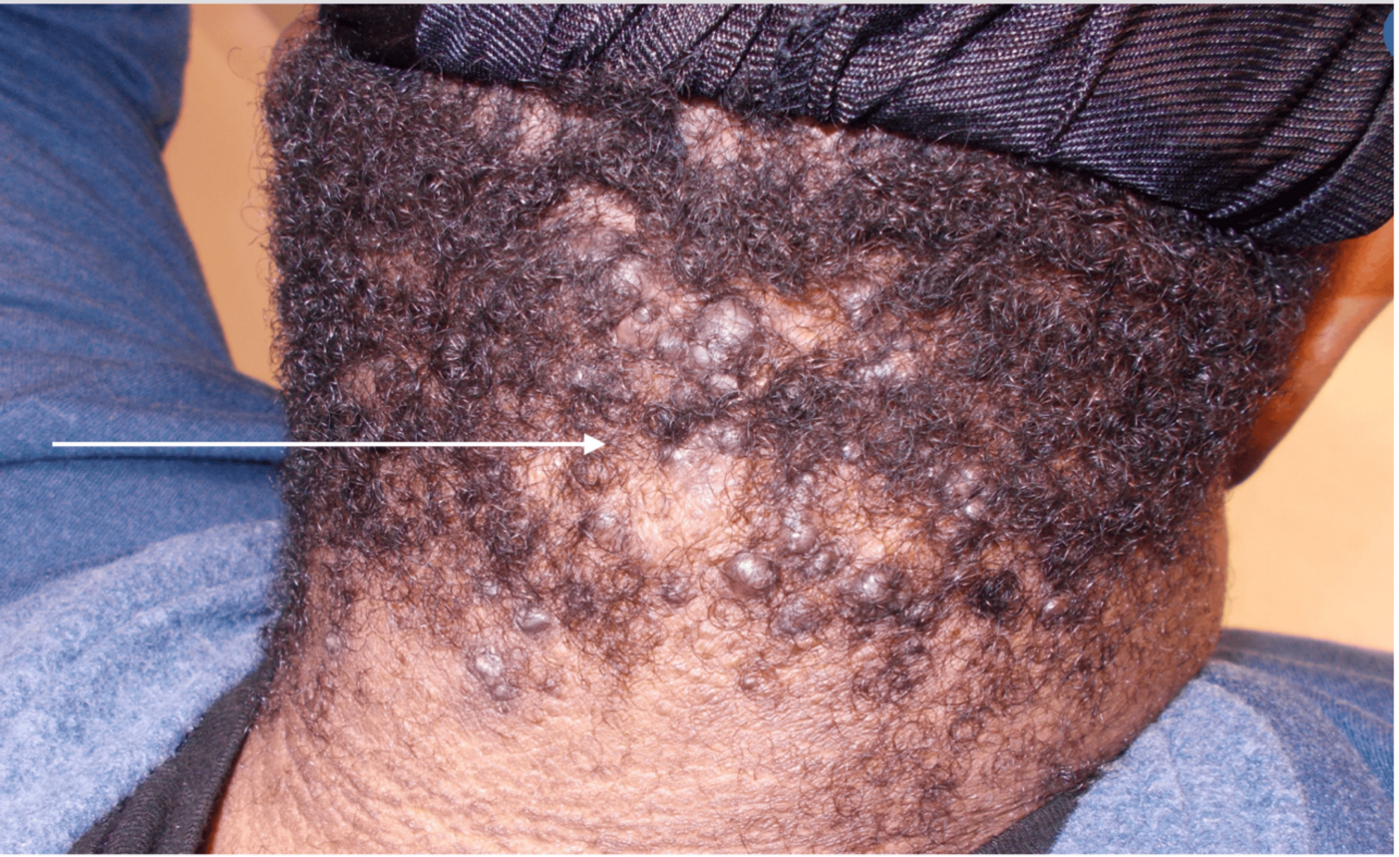
Acne keloidalis nuchae with cicatricial alopecia on first follow-up visit.

Hyperpigmented, velvety plaques were also present on the posterior neck, indicating the presence of acanthosis nigricans. The diagnosis of AKN was made clinically based on the patient's history and physical examination. The eruption was classified as stage three AKN based on the size and distribution of the rash. The laboratory analysis had already been performed by his primary care physician and did not reveal any abnormalities. The patient was initially started on topical therapy, including corticosteroids and an antimicrobial wash, as well as oral antibiotics with doxycycline. He was also instructed on lifestyle modification, including avoiding short haircuts and wearing hats. At his follow-up visit three months later, slight improvement was reported by the patient and seen on exam; however, areas of alopecia surrounding the lesions were also noticeable (Figure 2).

**Figure 3:**
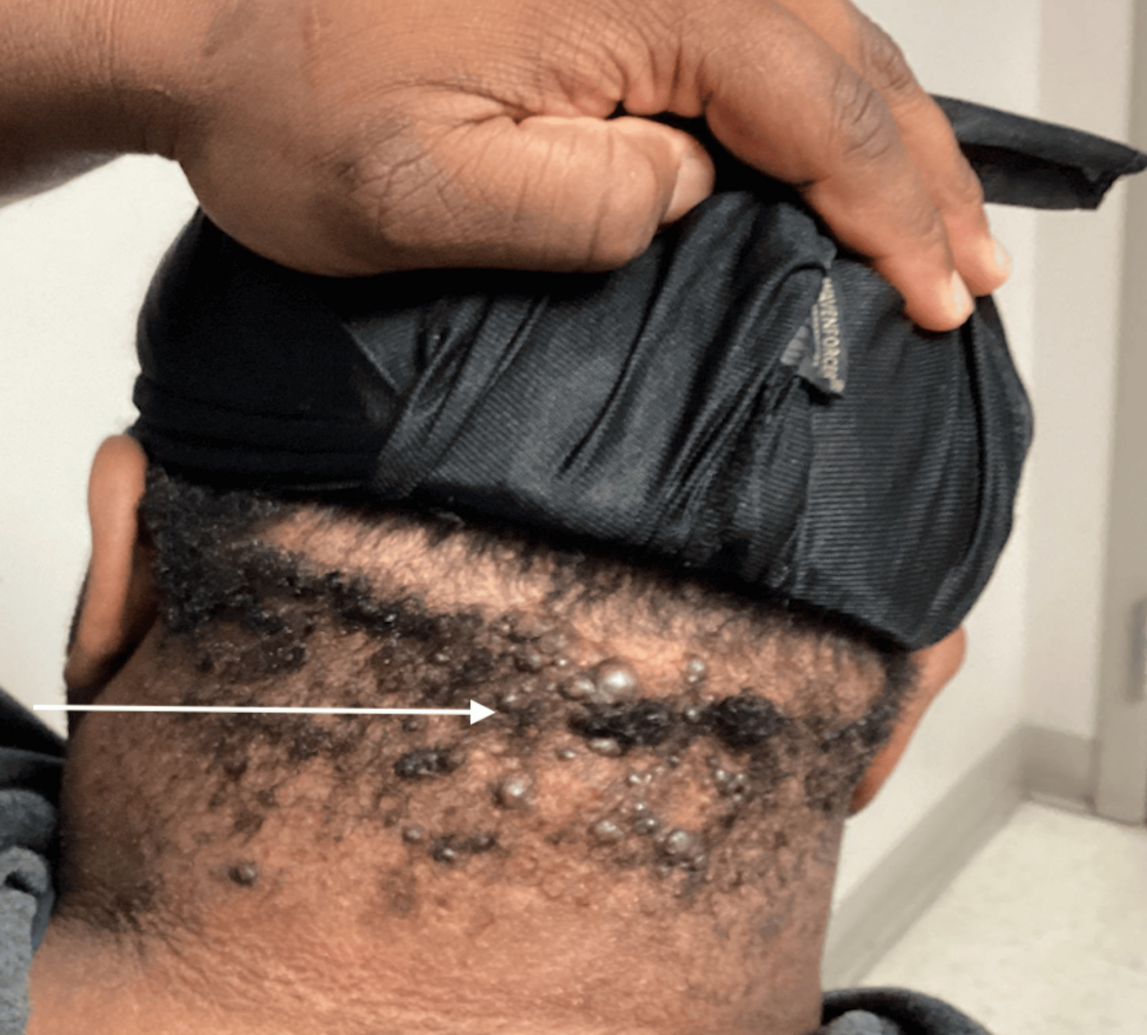
Acne keloidalis nuchae after multiple visits and interventions.

He continued to wear a do-rag, likely to hide the underlying lesions. He was then started on intralesional corticosteroids administered in the clinic. After inconsistent and only moderately effective treatments, due to the patient missing follow-up visits, and multiple failures of topical, intralesional, and oral therapies, it was determined that the patient may benefit from dermatology evaluation with consideration for laser therapy (Figure 3).

Oral isotretinoin was deemed contraindicated by ophthalmology due to his ongoing eye disease and studies that suggest a link between the use of oral isotretinoin and worsening keratoconus. The patient was unfortunately lost to follow-up.

## Discussion

The etiology of AKN is unknown; however, it is suggested that the disease process is due to chronic inflammation of the hair follicles due to a variety of predisposing or exacerbating factors. These factors range from close-shave haircuts, trauma, friction, heat, humidity, or infection. Other factors could include autoimmunity, excess androgens, seborrhea, or medication side effects (cyclosporin, lithium) [2,4].

AKN is most often found in people with Fitzpatrick skin phototypes V and VI, with a prevalence in the African American population of 0.5% to 13.6% [1,5]. It typically presents after puberty and affects males more than females by a ratio of 20:1 [1,2].

AKN occurs due to skin injury from repeated friction, trauma, or infection, which subsequently leads to an abnormal immune response [1]. This process causes scarring of the hair follicles, with the development of keloid-like scars and cicatricial alopecia [1].

A thorough history should include a review of the haircut routine, trauma, identification of triggers that can cause chronic friction and infection, and a review of medications [2]. The physical examination should be thorough, involving inspection and palpation of the nuchal and occipital scalp. Examination findings may include firm, dome-shaped, inflammatory papules and pustules, keloid-like papules and plaques, and scarring alopecia [2,3].

Comorbidities associated with AKN include several inflammatory scalp and general dermatologic conditions, such as chronic scalp folliculitis and hidradenitis suppurativa [6]. Importantly, AKN is also commonly comorbid with metabolic syndrome and its four components: diabetes mellitus, hypertension, obesity, and dyslipidemia [6]. As in the presented patient, acanthosis nigricans, a dermatologic manifestation of metabolic syndrome, is sometimes present with AKN. Evaluation may therefore include laboratory testing with a hemoglobin A1c and lipid panel [6].

A clinical diagnosis based on the history and physical examination is typically easily made. Although not frequently needed, histological analysis may vary depending on the time of the biopsy. Neutrophils and lymphocytes around the isthmus of the follicle and naked hair shafts in the dermis can be seen. As the disease progresses, destruction of the sebaceous glands, chronic granulomatous inflammation with collagen deposits, and fibrosis can be observed. If there is fluid discharge, sinus tracts may be present [2,7]. Microbiologic studies can also be obtained to identify the presence of any primary or secondary infectious process [2]. AKN can be classified by lesion distribution and morphology. This further helps classify the lesions into classes I-IV [8]. Class I lesions are less than 3 mm, confined to a sagittal width. Class II lesions are between 3 and 6.5 cm in size, confined to a sagittal width. Class III lesions are greater than 6.5 cm or breach the clinical demarcation lines, but generally are restricted to the nuchal area. Class IV lesions are widespread and exceed the nuchal area [8].

Treatment depends on the severity of the disease process; however, there is no optimal first-line therapy. The various management options should be provided to the patient, and shared decision-making between the patient and physician is typically preferred. However, it is essential to note that the overall treatment is somewhat difficult with unpredictable outcomes. Mild disease is treated with triamcinolone and topical retinoids for two to four weeks. Clindamycin solution is added if pustules are present [1]. Intralesional triamcinolone is an alternative treatment option for mild to moderate disease [1]. Topical antimicrobials, such as benzoyl peroxide or chlorhexidine, can help prevent secondary bacterial infections [2,9]. Other treatment options include oral isotretinoin, cryotherapy, oral doxycycline, phototherapy, laser therapy, and surgery [9]. Laser therapy, particularly with a 1064 nm Nd:YAG (neodymium-doped yttrium aluminum garnet) laser and an 810 nm diode laser, is most effective in improving the number and size of lesions [2,10]. Surgical excision is considered for extensive and/or refractory lesions; however, mild recurrences have been reported [11]. Maintenance therapy includes the use of topicals such as steroids, benzoyl peroxide, and retinoids [2].

Coexisting obesity has been linked to difficulty in managing AKN [12]. Deeper skin folds at the nape of the neck in obese patients may increase friction and irritation [12]. The heightened generalized inflammation associated with comorbid metabolic syndrome may also contribute to challenges in management [12]. Surgical interventions may be complicated by delayed wound healing and infection [12]. The surgical procedure itself may be more technically challenging in obese patients, as the increased vascularity associated with excess adipose tissue complicates dissection and closure [12].

Painful interventions such as cryotherapy, steroid injections, laser treatments, and surgery may be taxing on the patient and contribute to poor compliance with the management plan [2].

Some differential diagnoses include psoriasis, acne conglobata, acne vulgaris, acneiform eruptions, folliculitis decalvans, hidradenitis suppurativa, bacterial folliculitis, acne mechanica, tinea barbae, perifolliculitis capitis abscedens et suffodiens, or cancer [2,3].

The disease progression of AKN is slow, and there is no definitive cure. Early interventions help improve outcomes and potentially limit progression and scarring [3].

Complications such as recurrence and permanent scarring can occur [5]. The psychological impact can be distressing due to multiple factors, including its lifelong duration and cosmetic disfigurement [3]. AKN can be associated with metabolic syndrome, hidradenitis suppurativa, and pseudofolliculitis barbae [1].

Patient education is a vital step in management. Avoiding frequent close shaves, short haircuts, and picking and scratching can help prevent potential exacerbations [1,9]. Additionally, minimizing irritation from helmets, hats, or clothing can also help prevent potential exacerbations.

AKN often requires a multidisciplinary team for its management. It can be treated within the primary care setting; however, if laser or phototherapy is required, referral to dermatology would likely be necessary. Access to optimal management may be challenging due to the limited availability of specialty treatment centers and the lower socioeconomic status of those primarily affected. If surgical excision is deemed appropriate, consultation with a specialist is required. Lifestyle modification, such as weight loss, may be assisted by a dietitian or nutritionist. Optimizing care of comorbid metabolic conditions such as diabetes and dyslipidemia may improve outcomes. Psychotherapy should be considered in patients at risk for mental health disorders due to the psychological impact of the disease process.

## Conclusions

AKN, a chronic inflammatory skin condition involving the hair follicle, can ultimately lead to keloid-like scarring and permanent alopecia. It is a skin condition found in people of color, particularly Fitzpatrick skin phototypes V and VI. Identifying and treating this condition early on can lead to the prevention of progression and irreversible scarring. Although various treatments are available, including topical and systemic therapies, intralesional corticosteroids, cryotherapy, laser therapies, and surgical excision, outcomes are often suboptimal, and recurrences are frequent. Poor outcomes may be secondary to the availability of specialty interventionalists and the cost of effective treatment strategies such as laser therapy. The disproportionately high incidence of obesity and metabolic syndrome in communities of color predominantly affected by AKN may also contribute to treatment difficulties. Poor compliance may be attributed to the pain and discomfort associated with many of the therapeutic options, such as cryotherapy, injections, lasers, and surgical intervention. This case report aids in recognizing this disease process in a patient population that is underrepresented in dermatology textbooks and clinical research, so that early interventions can be implemented to avoid poor outcomes. Continuing investigations into management strategies and barriers to effective treatment are warranted to address this pervasive syndrome.
